# Influence of Extraction Conditions on Ultrasound-Assisted Recovery of Bioactive Phenolics from Blueberry Pomace and Their Antioxidant Activity

**DOI:** 10.3390/molecules23071685

**Published:** 2018-07-11

**Authors:** Bio Sigui Bruno Bamba, John Shi, Carole C. Tranchant, Sophia Jun Xue, Charles F. Forney, Loong-Tak Lim

**Affiliations:** 1 Department of Biochemistry and Genetics, Biological Sciences Training and Research Unit, Université Peleforo Gon Coulibaly, Korhogo BP 1328, Côte d’Ivoire; 2 Agriculture and Agri-Food Canada, Guelph Research and Development Centre, Guelph, ON N1G 5C9, Canada; jun.xue@agr.gc.ca; 3 School of Food Science, Nutrition and Family Studies, Université de Moncton, Moncton, NB E1A 3E9, Canada; 4 Agriculture and Agri-Food Canada, Kentville Research and Development Centre, Kentville, NS B4N 1J5, Canada; charles.forney@agr.gc.ca; 5 Food Science Department, University of Guelph, Guelph, ON N1G 2W1, Canada; llim@uoguelph.ca

**Keywords:** blueberry pomace, polyphenols, flavonoids, anthocyanins, antioxidant activity, ultrasound-assisted extraction, extraction parameters, green technology

## Abstract

The increase in diet-related chronic diseases has prompted the search for health-promoting compounds and methods to ensure their quality. Blueberry pomace is a rich yet underutilized source of bioactive polyphenols. For these high-value bioactive molecules, ultrasound-assisted extraction (USAE) is an attractive and green alternative to conventional extraction techniques for improving purity and yields. This study aimed to assess the impact of USAE parameters (sonication time, solvent composition, solid/liquid ratio, pH and temperature) on the recovery of phenolic compounds from blueberry pomace and antioxidant activity of the extracts. Total phenolic, flavonoid and anthocyanin contents (TPC, TFC and TAC) and 2,2-diphenyl-1-picrylhydrazyl (DPPH) free radical scavenging activity were analysed. USAE in 50% ethanol/water was the most efficient, yielding the highest TPC (22.33 mg/g dry matter (DM)), TFC (19.41 mg/g DM), TAC (31.32 mg/g DM) and DPPH radical scavenging activity (41.79 mg Trolox/g DM). USAE in water showed the lowest values even at low (1/40) solid/liquid ratio (7.85 mg/g DM, 3.49 mg/g DM, and 18.96 mg/g DM for TPC, TFC and TAC, respectively). Decreasing the solid/liquid ratio in water or 50% ethanol significantly increased TPC, TFC, TAC and DPPH radical scavenging. With ethanol, increasing the temperature in the range 20–40 °C decreased TPC but increased TFC and DPPH radical scavenging activity. Anthocyanin profiles of water and ethanolic extracts were qualitatively similar, consisting of malvidin, delphinidin, petunidin and cyanidin. These findings indicate that USAE is a method of choice for extracting high-value bioactive phenolics from blueberry pomace. Selective enrichment of different phenolic fractions is possible under select extraction conditions.

## 1. Introduction

Polyphenols are natural secondary metabolites produced by plants. They are classified into different groups as phenolic acids (derivatives of benzoic acid and cinnamic acid), flavonoids (flavonols, flavones, isoflavones, flavanones, anthocyanidins and flavanols (e.g., catechins and proanthocyanidins)), stilbenes and lignans [1]. Polyphenols are known for their strong antioxidant properties and potential health benefits, including the prevention of chronic illnesses such as cardiovascular diseases, type 2 diabetes, osteoporosis, neurodegenerative diseases and some cancers, although their protective action goes beyond the modulation of oxidative stress [2]. They are increasingly used as nutritional supplements, nutraceuticals, as well as ingredients in foods, functional foods, pharmaceutical and cosmetic products.

Berries such as blueberries (*Vaccinium* section *Cyanococcus* spp.) contain abundant phenolic compounds, including anthocyanins (derived from anthocyanidins by glycosylation), flavonols and chlorogenic acids, which are mainly found in berry skin [3,4,5]. Some of these compounds are pigments that impart pleasant and characteristic colours to the fruits. Berry fruits can be processed into juice, wine, jam and marmalade, among other foods. Berry processing generates large quantities of pomace, which consists of skin, seeds and some flesh [6,7,8]. Berry flesh contains about 10% of the total polyphenols, while the skin and seeds contain 28–35% and 60–70%, respectively, which makes berry processing by-products an excellent source of polyphenols [9]. According to Struck et al. [10], processing berries into juice leaves approximately 20–30% of the fruit as pomace. Blueberry production in Canada, the second largest producer worldwide after the United States, reached 176,641 tons in 2017 [11], with some consumed fresh and some being processed. Thus, blueberry pomace from food processing results in considerable losses in polyphenols and other valuable bioactive phytochemicals (most notably, carotenoids, vitamins and dietary fiber) if these are not recovered. Extracting these compounds from the pomace for subsequent use in foods, pharmaceuticals or fine chemicals for healthcare and lifestyle applications is considered the best approach for maximal valorisation of this by-product.

With increased awareness of food additives, functional foods and sustainable food production in recent years, consumers have become more demanding in regard to food quality. This promotes a high demand for more natural and safe sources of ingredients. Fruits, vegetables and their by-products are prime sources for the recovery of natural polyphenols with multiple functionalities. Several extraction techniques are available but the conventional ones (e.g., decoction, digestion, infusion, maceration, percolation, Soxhlet extraction, hot continuous extraction and counter-current extraction) have notorious drawbacks. They tend to be laborious, time consuming, produce diluted extracts, cause degradation of some of the desired compounds, and involve large amounts of solvents which contribute to environmental pollution and greenhouse effect. The remaining solvent residues are often flammable, volatile and toxic [12,13,14,15]. For safety, environmental and economical sustainability, green or eco-friendly processes are being developed using various methods such as microwave-assisted extraction, supercritical fluid extraction, accelerated solvent extraction, enzyme-assisted extraction and ultrasound-assisted extraction (USAE) [16,17]. Their main advantages include shorter extraction times, reduced energy consumption, fewer negative environmental impacts, increased safety as well as enhanced innovation and competitiveness [18], all of which contribute to improving the sustainability of the value chain that supplies the extracts. 

In this context, USAE is a particularly attractive method due to effective extraction, energy saving and the use of moderate temperatures, which is beneficial for heat-sensitive compounds [19]. It is thus widely used to extract bioactive compounds from plant materials [20]. The main drawback of USAE is the unavoidable use of organic solvents in some applications, yet the equipment is simpler and the overall cost is lower compared to supercritical CO_2_ extraction which does not use organic solvent [21]. Still, this limitation can be overcome by using ethanol as USAE solvent as it is safe to use in food systems, completely biodegradable, available in high purity form and at low price [14]. Several USAE parameters affect the quality of the extracts. Among them, sonication time, temperature, solvent composition, solid/solvent ratio, particle size of the raw material, matrix parameters as well as ultrasonic irradiations (power, frequency) can affect the quantity, composition and biochemical properties of the extracts [12,19,20,22].

Several studies have examined the USAE of bioactive phytochemicals. The results tend to differ markedly among studies according to operating conditions. Moreover, each plant material has its own unique properties in terms of chemical composition, physical characteristics, processing, storage conditions, origin (e.g., genetics and growing environment) and provider, for instance [13], which seem to affect the outcomes of USAE. Although the extraction of berry polyphenols by USAE has been studied quite extensively, extraction parameters vary widely. There is no consensus on the optimum USAE parameters and, because of the extremely diverse nature of polyphenolics and biological matrices in which they are embedded, the extraction of these compounds cannot be easily standardized or generalized [9]. Therefore, USAE methods must be developed to be suitable for use with the plant material considered and the phenolic compounds or fractions of interest. In order to develop an extraction method well tailored for blueberry pomace, the aim of the present study was to assess the effects USAE parameters (sonication time, solid/liquid ratio, solvent composition, pH and extraction temperature) on the total phenolic, flavonoid and anthocyanin contents and total antioxidant activity of extracts prepared from blueberry wine pomace.

## 2. Results and Discussion

This study is the first to investigate the influence of USAE conditions on the recovery of phenolic compounds from blueberry pomace and antioxidant activity of the extracts. The parameters that were varied are sonication time, solid/liquid ratio, solvent composition (% ethanol in water), pH and temperature. The corresponding extraction conditions are presented in Table 1. Five series of experiments were conducted to assess the influence of each parameter and identify suitable levels for each parameter.

### 2.1. Effect of Sonication Time on the Phenolic Contents of Blueberry Pomace Water Extracts

**Figure 1:**
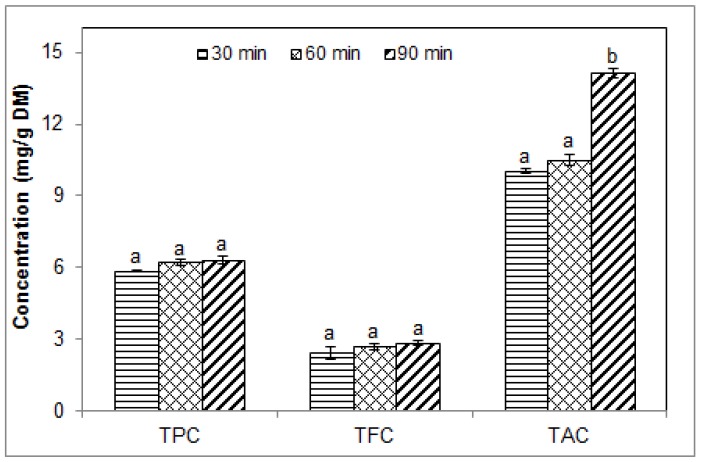
Effect of sonication time on the phenolic contents of blueberry pomace water extracts from ultrasound-assisted extraction at 40 °C. TPC, TFC, TAC: total phenolic, flavonoid and anthocyanin contents, respectively. Means (*n* = 3 replicates) ± standard deviation (SD). Different letters indicate significant effect (*p* ≤ 0.05).

The effect of sonication time (from 30 to 90 min) was assessed using water as the extraction solvent with a solid/liquid ratio of 1/20 at 40 °C. The results are shown in Figure 1. Overall, the water extracts of blueberry pomace contained relatively small amounts of phenolics, below 15 mg/g of dry matter (DM). Over the time range investigated, total phenolic content (TPC in gallic acid equivalents) ranged from 5.84 ± 0.03 to 6.31 ± 0.15 mg GAE/g DM, total flavonoid content (TFC, catechin equivalents) ranged from 2.45 ± 0.25 to 2.85 ± 0.11 mg CE/g DM, while total anthocyanin content (TAC, malvidin equivalents) ranged from 10.04 ± 0.10 to 14.1 ± 0.15 mg ME/g DM. Extraction duration significantly affected TAC, but not TPC and TFC. TAC after extraction for 90 min was significantly higher than after 30 and 60 min.

These relatively low concentrations can be attributed to the physicochemical properties of polyphenols. These compounds contain several nonpolar portions, including the aromatic rings, which limit their solubility and thus their extraction in a highly polar solvent such as water. The higher levels of TAC overall may be explained by greater solubility in water due to the positive charge of anthocyanins and the presence of more hydroxyl groups in these molecules. Greater solubility would enhance the mass transfer which governs the extraction process. The gradual enhancement of TAC content after 60 min as ultrasound-assisted extraction progressed is consistent with a two-stage extraction process.

Our findings are consistent with previous reports despite obvious differences in extraction conditions and plant materials. Low concentrations of TPC and TFC (6.25 mg GAE/g and 4.04 mg quercetin equivalents/g, respectively) were reported by Do et al. [23] using simple water extraction (without sonication) of *Limnophila aromatic* for 20 min. They suggested that these low levels could be due to the ability of water to extract more nonphenolic substances such as carbohydrates, or to the formation of complexes involving phenolic compounds which may decrease their solubility in water. Wang et al. [24] found no increase in TPC and TFC with extraction time beyond 15 min when extracting blueberry leaves using ultrasound-negative pressure cavitation extraction. With USAE of dried chokeberries in 50% ethanol, Ćujić et al. [25] reported no difference between the TPC obtained after 30 and 60 min. TAC, however, was significantly greater after 60 min than after 30 min. With maceration by simple diffusion (no sonication) in an ethanolic solvent, they found that extraction times of 30–90 min (for TPC) and 60–90 min (for TAC) yielded higher contents compared to shorter durations [25]. It is noteworthy that longer extraction times in water can lead to decrease in TPC and TAC, as shown by Lapornik et al. [26] with red currant and black currant by-products (marc) extracted for 1 to 24 h in water without sonication. This indicates that with some plant materials, excessive extraction duration in water may cause degradation of some target compounds resulting in reduced contents. Although this was not observed in our work using USAE in water, we concluded that extraction times between 30 and 60 min would be a good compromise in order to avoid longer processing times. When higher TAC are desired, sonication could be extended to 90 min. In our work, the effect of sonication time was investigated only with water. Because of this limitation, it is not certain whether similar effects would be observed with ethanol. Extraction times of 60 min and 40 min were used in subsequent experiments.

### 2.2. Effect of Solid/Liquid Ratio on the Phenolic Contents and Antioxidant Activity of Blueberry Pomace Extracts

**Figure 2:**
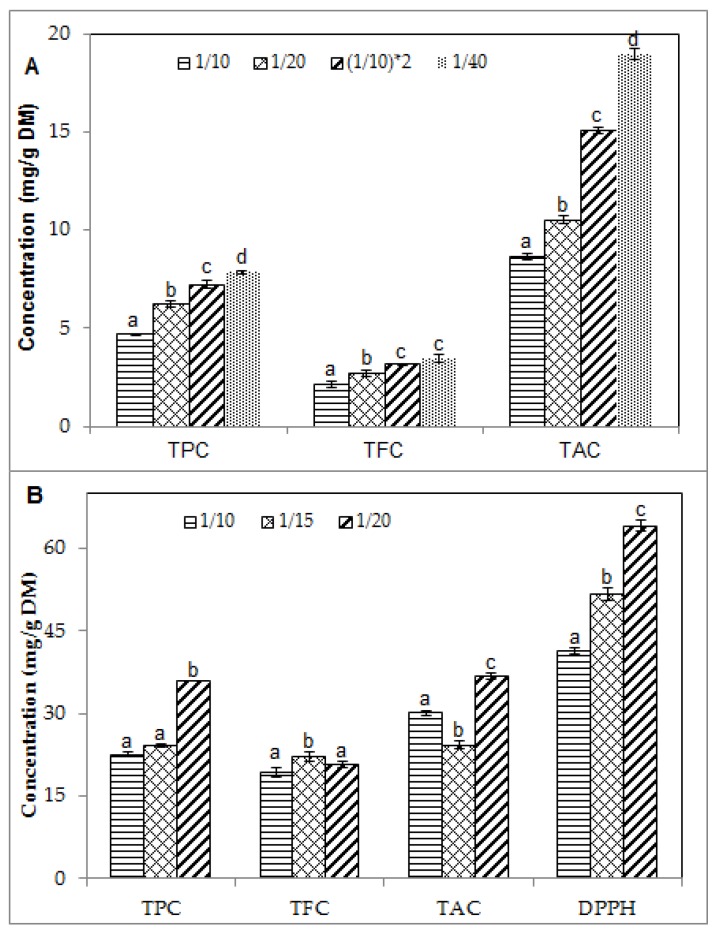
Effect of solid/liquid ratio on the phenolic contents and antioxidant activity of blueberry pomace (**A**) water extracts and (**B**) 50% ethanolic extracts from ultrasound-assisted extraction (40 °C/60 min). TPC, TFC, TAC: total phenolic, flavonoid and anthocyanin contents, respectively; DPPH: antioxidant activity by DPPH assay. Means (*n* = 3 replicates) ± SD. Different letters indicate significant effect (*p* ≤ 0.05).

The effect of solid/liquid ratio on TPC, TFC and TAC of the blueberry extracts from USAE was assessed at 40 °C using water and 50% ethanol as the extraction solvents and varying the solid/liquid ratio from 1/10 to 1/40 and from 1/10 to 1/20, respectively. Extraction duration was 60 min. Total antioxidant activity of the ethanolic extracts was evaluated using the 2,2-diphenyl-1-picrylhydrazyl (DPPH) essay. With both solvents, TPC, TFC and TAC increased significantly with decreasing the solid/liquid ratio, as shown in Figure 2. This is consistent with the fact that lower solid/liquid ratios increase the contact surface between the plant material and the solvent, which enhances the mass transfer of soluble compounds from material to solvent [27,28]. With water (Figure 2A), TFC levels remained low, even as water quantity was multiplied by four. This may be due to low solubility of these compounds in water. The highest value of TFC was 3.49 ± 0.19 mg CE/g DM at 1/40. Comparison of the phenolic contents obtained at 1/20 and (1/10) × 2 shows that two sequential water extractions using a solid/water ratio of 1/10 for 30 min each time, while maintaining the total extraction time constant (60 min), significantly increased the extraction yield of all the phenolic fractions considered. This beneficial effect can be explained by the renewed concentration gradient between the plant material and the solvent, which occurs after renewing the extraction solvent and results in enhanced mass transfer.

With 50% ethanol (Figure 2B), TPC and DPPH free radical scavenging activity increased continuously with decreasing solid/liquid ratio from 1/10 to 1/20. The corresponding values were 22.57 ± 0.53, 24.16 ± 0.25 and 35.95 ± 0.12 mg GAE/g DM for TPC and 41.39 ± 0.61, 51.75 ± 1.21 and 64.25 ± 0.39 mg TE/g DM for DPPH. This suggests that the antioxidant activity of the extracts depends on their concentration in total phenolic compounds. For TFC and TAC, a slightly different trend was observed as the increase in TFC and TAC was not continuous across all the values of solid/liquid ratio. These findings are consistent with the increased TPC and TAC reported by Ćujić et al. [25] using maceration of chokeberries in an ethanolic solvent. In their work, the solid/liquid ratio varied from 1/10 to 1/30 and the increase in TPC and TAC levelled off between 1/20 and 1/30, with TAC seemingly declining at 1/30 albeit not significantly. This suggests that excessive dilution of the plant material may not lead to further enhancement of TAC under the conditions investigated. Excess of solvent, without beneficial increase in phenolic contents, should also be avoided as it leads to solvent wastage and unwarranted increase of operating costs [24]. In the present study with USAE and blueberry pomace, a ratio of 1/15 in 50% ethanol was found to be a suitable compromise and was used in subsequent experiments.

Across the range of solid/liquid ratios investigated, superior concentrations of phenolic compounds were achieved in 50% ethanol. As shown in Figure 2, TPC, TFC and TAC in the ethanolic extracts were about 5, 3 and 1.5 times greater on average, respectively, than in the water extracts. Even at the lowest solid/liquid ratio (1/40) in water, TPC, TFC and TAC remained lower than those obtained with 50% ethanol at the highest solid/liquid ratio (1/10). Higher concentrations of TPC and TAC in ethanol compared to water have been reported by Ćujić et al. [25] (50% ethanol) and Lapornik et al. [26] (70% ethanol), although their work did not involve sonication. The magnitude of the difference in phenolic contents between ethanolic and water extracts was about 1.5–2 times in the former study, which was conducted with macerated chokeberries [25], while in the latter study, it was about 2 times for macerated red and black currant residue (marc) and 5–10 times for macerated grape marc [26]. This indicates that the extent of the beneficial effect of ethanolic solvents on extraction yields varies greatly with plant material and extraction conditions. Binary solvents of ethanol and water present several advantages as ethanol can enhance the solubility of some solutes such as polyphenols, while water increases their desorption from plant matrices [29]. Differences in the structure of phenolic compounds affect their solubility in solvents of different polarities [30]. The addition of water to ethanol and other organic solvents generally creates a more polar medium, which facilitates the extraction of polyphenols [31].

### 2.3. Effect of Ethanol Concentration on the Phenolic Contents and Antioxidant Activity of Blueberry Pomace Extracts 

**Figure 3:**
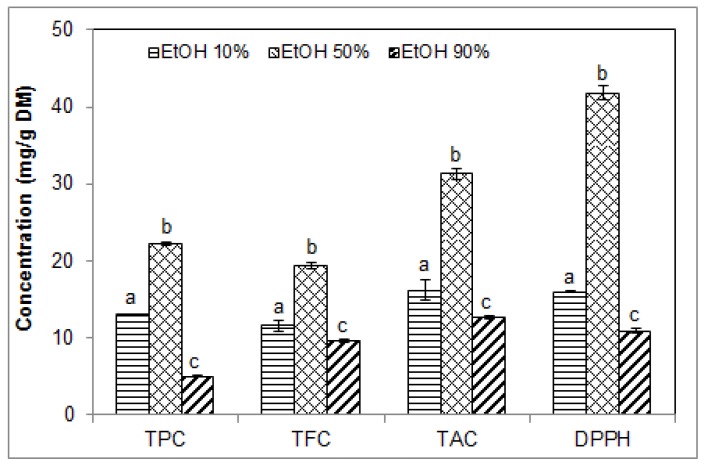
Effect of ethanol concentration in the extraction solvent on the phenolic contents and antioxidant activity of blueberry pomace ethanolic extracts from ultrasound-assisted extraction (40 °C/40 min). TPC, TFC, TAC: total phenolic, flavonoid and anthocyanin contents, respectively; DPPH, antioxidant activity by DPPH assay. Means (*n* = 3 replicates) ± SD. Different letters indicate significant effect (*p* ≤ 0.05).

To maximize the extraction of blueberry pomace phenolic compounds by USAE, ethanol concentration was varied between 10% and 90% in water. Extraction was performed at 40 °C for 40 min with a solid/liquid ratio of 1/15 as determined in our previous sets of experiments. Ethanol was used as co-solvent as it is known to be a suitable solvent for polyphenol extraction and is recognized as safe for use in food and pharmaceutical applications intended for humans. Significantly higher TPC, TFC, TAC and DPPH free radical scavenging activity were found with 50% ethanol compared to 10% and 90% ethanol, as illustrated in Figure 3. Increasing the concentration of ethanol from 50% to 90% resulted in values that were significantly lower than those obtained with 50% and 10% ethanol, and similar to those obtained with water alone (Figure 1). Maximum and minimum values obtained with 50% vs. 90% ethanol were 22.23 ± 0.15 vs. 5.02 ± 0.09 mg GAE/g DM for TPC, 19.41 ± 0.33 vs. 9.71 ± 0.19 mg CE/g DM for TFC, and 31.32 ± 0.73 vs. 12.75 ± 0.17 mg ME/g DM for TAC, and 41.79 ± 0.92 vs. 10.95 ± 0.28 mg TE/g DM for antioxidant activity.

These findings concur with those of Ćujić et al. [25] who reported greater TPC and TAC (18.2 mg GAE/g DM and 0.21%, respectively) with 50% ethanol than with 70% and 96% ethanol upon the maceration of chokeberries. With 96% ethanol, these values were drastically reduced. In contrast, Dent et al. [32], also using maceration, found that an increase in the volume fraction of ethanol or acetone in water above 30% and up to 70% resulted in a considerable drop of extraction efficiency of total polyphenols from *Salvia officinalis*. Safdar et al. [29], on the other hand, reported significantly higher TPC with 80% ethanol compared to 50% and 100% ethanol (24, 22 and 20 mg GAE/g, respectively) using USAE of *Citrus reticulate* polyphenols. At all three ethanol concentrations, TPC values in their work were similar to the value we obtained using 50% ethanol. Wang et al. [24] suggested that the range of 60–70% ethanol is the most suitable for ultrasound-negative pressure cavitation extraction of polyphenols from blueberry leaves. They found that TPC and TFC were increased when increasing ethanol concentration from 40% to 70%, while further increase of ethanol concentration up to 90% decreased the extraction yield. 

These variable and sometimes conflicting reports confirm that the efficiency of polyphenol extraction in ethanolic solvents is greatly influenced by the plant material and the overall extraction conditions. Despite these variations, most studies indicate that too high or too low concentrations of ethanol in water are not conducive to the simultaneous extraction of all the phenolic compounds. Ethanol reduces the dielectric constant of the aqueous solvent, thus increasing the diffusion of molecules such as polyphenols in the solvent, but too high concentrations of ethanol could dehydrate the plant cells, which could hinder the diffusion of polyphenols from the plant material to the solvent [33]. Under the USAE conditions used in the present work with blueberries pomace, a concentration of 50% ethanol was highly effective for extracting total phenolics, flavonoids and anthocyanins. The superior antioxidant activity obtained with 50% ethanol can be attributed to the superior phenolic contents of these extracts. It cannot be ruled out that an ethanol concentration above 50%, but lower than 90%, could also be effective, but 50% seems a good compromise in order to keep the costs down and the extraction method as environmentally friendly as possible.

### 2.4. Effect of pH on the Phenolic Contents and Antioxidant Activity of Blueberry Pomace Extracts

**Figure 4:**
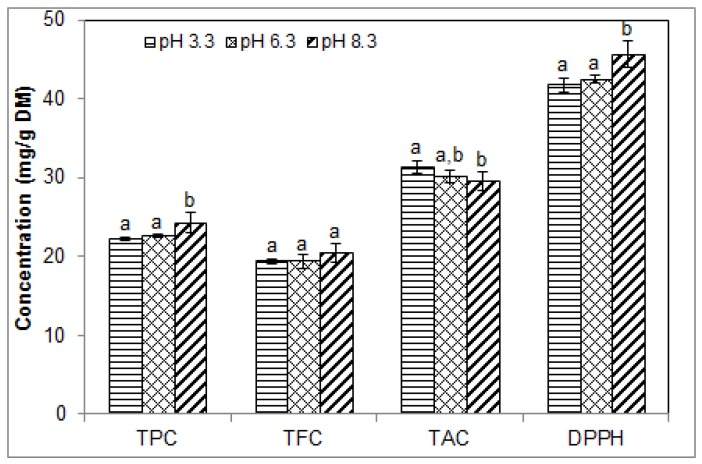
Effect of pH of the extraction solvent on the phenolic contents and antioxidant activity of blueberry pomace ethanolic extracts from ultrasound assisted-extraction (50% ethanol, 40 °C/40 min). TPC, TFC, TAC: total phenolic, flavonoid and anthocyanin contents, respectively; DPPH, antioxidant activity by DPPH assay. Means (*n* = 3 replicates) ± SD. Different letters indicate significant effect (*p* ≤ 0.05).

The effect of solvent pH (from 3.3 to 8.3) was assessed using 50% ethanol in water with a solid/liquid ratio of 1/15. Extraction was conducted at 40 °C for 40 min as in the previous set of experiments. TPC and antioxidant activity were both significantly increased when the pH was increased above 6.3, while TAC was significantly decreased above this value (Figure 4). The corresponding values at pH 3.3 vs. 8.3 were 22.23 ± 0.15 vs. 24.28 ± 0.27 mg GAE/g DM for TPC, 31.31 ± 0.44 vs. 29.58 ± 0.27 mg ME/g DM for TAC, and 41.78 ± 0.98 vs. 45.65 ± 1.74 mg TE/g DM for antioxidant activity. There was no statistically significant difference in TFC values (19.41 ± 0.33 vs. 20.50 ± 1.20 mg CE/g DM) in the pH range investigated. These findings are in agreement with those shown in Figure 2B and Figure 3, which suggest that the antioxidant activity of the ethanolic extracts depends mainly on their total phenolic content.

These findings support some previous reports but discrepancies were also noted. After sonication of blueberry leaves in 50% ethanol for 60 min at pH 2 and 6, Cheng et al. [34] found lower contents of total extractable polyphenols at pH 6 than at pH 2, which seems to contradict our results for TPC. However, they found higher contents of non-extractable polyphenol at pH 6 [34]. At pH 6, the extractable anthocyanins were about two-fold lower than at pH 2, which is consistent with our findings of lower TAC at pH 8.3 compared to pH 3.3. Lower TAC at basic pH values could indicate lower extraction or some degradation of the anthocyanins under basic pH conditions. Our findings and previous findings such as Cheng et al.’s are difficult to compare because the pH ranges were different; some analytical methods and extraction conditions were also different, as were the blueberry products subject to extraction (blueberry leaves vs. pomace).

Consistent with our results for TAC, Kalt et al. [35] reported a higher concentration of monomeric anthocyanins at pH 1, followed by pH 4 and finally pH 7. These were extracted from blueberry juice. Unlike in our work, however, they found that TPC were higher at pH 1 than at pH 4 and 7. They attributed the lower TPC at pH 4 and 7 to the irreversible loss of some anthocyanins and suggested that higher contents of polymeric anthocyanins at pH 4 and 7 may be due to greater self-association of anthocyanin molecules at high pH. For TAC and antioxidant activity, our findings concur with those of Librán et al. [33] who found a reduction of TAC between pH 2 and 12, and an increase in antioxidant activity between pH 2 and pH 5, 8 and 12. For TPC, however, they found decreased contents between pH 2 and 12. They suggested that the degree of correlation between antioxidant activity and phenolic contents depends not only on TPC, but also on the composition of the extracts. They used 50% ethanol to macerate grape wastes (1/25 *w*/*v*) at room temperature for 2 h [33]. For TPC and DPPH, our findings concur in part with those of Ruenroengklin et al. [36] who found increased TPC between pH 2 and 4 and increased DPPH free radical scavenging activity between pH 3 and 5. They also showed that TPC started to decline at pH 5 and 6, and that DPPH declined at pH 7, which was not observed in the present study, most likely because of differences in plant materials, constitutive polyphenols and extraction conditions. Ruenroengklin et al. [36] used litchi fruit pericarp tissue macerated in 60% ethanol. TPC and antioxidant activity from grape extracts were found to be stable during storage at pH 3 and 5, but declined with storage time at pH 7 and 9 [37].

Our findings and the available literature indicate that pH is an important parameter affecting the extractability of polyphenolic compounds. Different phenolic fractions seem to be affected differently, which can be used to selectively enrich the extracts in specific phenolic fractions. Proper adjustment of the pH can also help stabilizing these compounds. With USAE of blueberry pomace polyphenols, higher pH values in the range 6.3 to 8.3 were beneficial for enhancing TPC and antioxidant activity, but they decreased TAC.

### 2.5. Effect of Temperature on the Phenolic Contents and Antioxidant Activity of Blueberry Pomace Extracts

**Figure 5:**
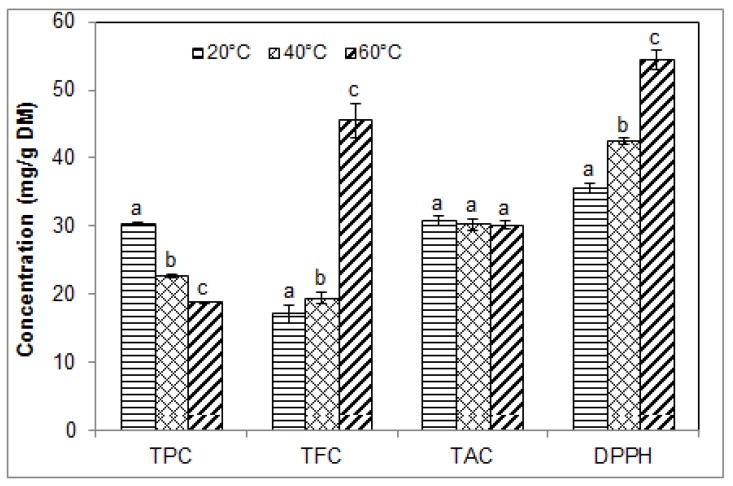
Effect of temperature on the phenolic contents and antioxidant activity of blueberry pomace ethanolic extracts from ultrasound assisted-extraction (50% ethanol, 40 min). TPC, TFC, TAC: total phenolic, flavonoid and anthocyanin contents, respectively; DPPH, antioxidant activity by DPPH assay. Means (*n* = 3 replicates) ± SD. Different letters indicate significant effect (*p* ≤ 0.05).

Increasing temperature generally accelerates reaction processes, including extraction and degradation. Beneficial effects of temperature during extraction processes are generally due to higher mass transfer rate, which leads to higher molecular diffusion [24], but an appropriate balance must be achieved to avoid degradation of heat-sensitive bioactives. In our study, the effect of extraction temperature was studied using 50% ethanol with a solid/solvent ratio of 1/15. USAE was conducted for 40 min at a pH of 3.3 in order to stabilise the anthocyanins as determined in our previous sets of experiments. As shown in Figure 5, when temperature was increased from 20 °C to 60 °C, TPC decreased significantly from 30.33 ± 0.27 to 18.74 ± 0.13 mg GAE/g DM. Conversely, TFC increased significantly from 17.05 ± 1.23 to 19.38 ± 0.86 mg CE/g DM between 20 °C and 40 °C, and then to 45.45 ± 2.46 mg CE/g DM at 60 °C. DPPH free radical scavenging activity also increased significantly from 35.59 ± 0.67 to 54.44 ± 1.36 mg TE/g DM at 20 °C and 60 °C, respectively. Since TAC remained unchanged (30.82 ± 0.79 to 30.12 ± 0.60 mg ME/g DM), increased antioxidant activity may be related to increased TFC.

Decreased TPC at higher extraction temperature may be explained by increased solvent vapour pressure and decreased surface tension as temperature increases, which affect cavitation bubble formation and collapse during sonication. At higher temperature, higher vapour pressure causes more solvent vapours to enter the bubble cavity and more numerous cavitation bubbles, but these collapse with less intensity, thus causing less cell disruption and reducing sonication effects [20]. A contributing factor could the degradation of some phenolic compounds at 40 °C and 60 °C, possibly due to hydrolysis, internal redox reactions or polymerization [32]. 

Although TPC declined at higher temperatures, its value at 60 °C (18.74 ± 0.13 mg GAE/g DM) was similar to that reported by He et al. [5] (16.01 ± 0.03 mg GAE/g) following USAE of blueberry wine pomace phenolics at 60 °C for 35 min in 70% ethanol (1/22 solid/liquid ratio). In their work, TPC increased between 50–60 °C, then decreased between 60–70 °C [5]. Likewise, Wang et al. [24] found a slight increase in TPC between 30–50 °C, followed by a slight decrease between 50–80 °C, when using ultrasound-negative pressure cavitation extraction of blueberry leaves for 15 min. In Kaderides et al. [31] study with USAE of pomegranate peels for 5 min, TPC increased between 25 °C and 35 °C, then declined between 35 °C and 45 °C. With maceration of *S. officinalis* leaves in 50% ethanol for 30 min to 90 min, decreased TPC was reported between 60 °C and 90 °C. [32]. Thus, available evidence indicates that optimal USAE temperature needs to be adapted to the plant material and phenolic fractions of interest. Other extraction parameters (e.g., sonication time and pH) also ought to be considered.

### 2.6. Anthocyanin Profiles in Water Extracts and Ethanolic Extracts from Blueberry Pomace

**Figure 6:**
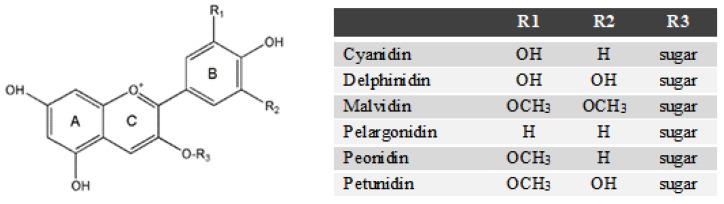
Structures of select anthocyanins adapted from [38]. All were detected in the water extracts and ethanolic extracts of blueberry pomace, except pelargonidin and peonidin.

Anthocyanins, which are the glycosylated derivatives of anthocyanidins, are one of the main phenolic fractions present in blueberries. They are natural pigments responsible for the blue-purple coloration of the berries. The structures of common anthocyanins are illustrated in Figure 6.

**Figure 7:**
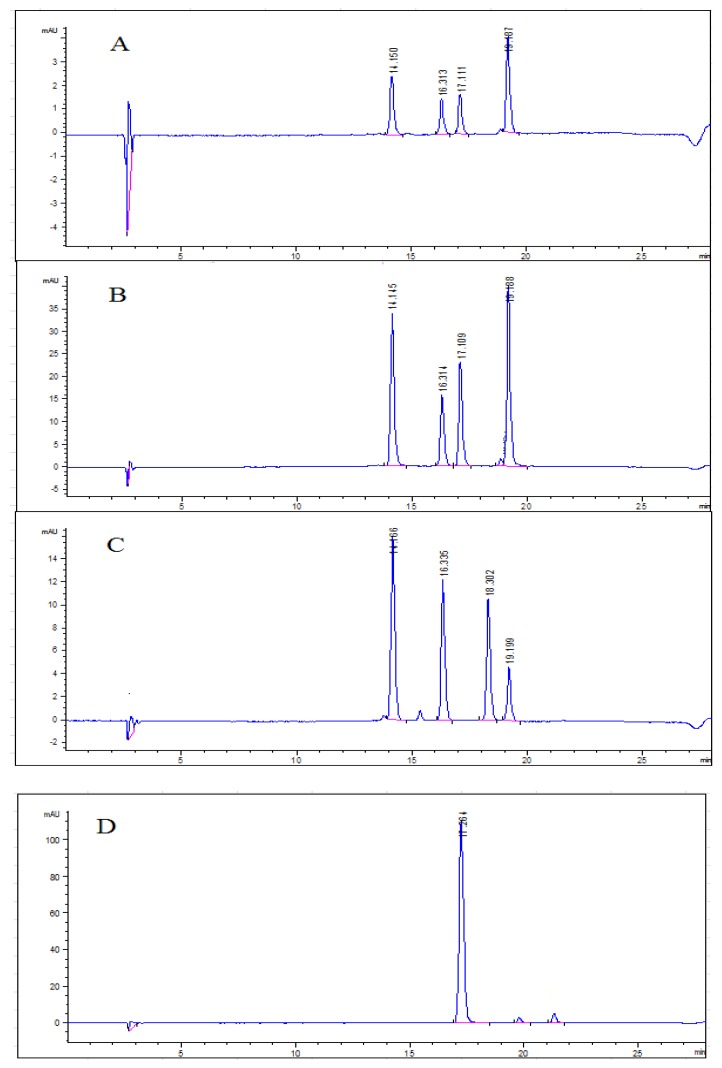
Representative high-performance liquid chromatography with photodiode array detector (HPLC–PAD) chromatograms of anthocyanidins in (**A**) blueberry pomace water extracts and (**B**) 50% ethanolic extracts, and in preparations from commercial standards: (**C**) delphinidin-cyanidin-pelargonidin-malvidin and (**D**) petunidin. Approximate elution times: delphinidin 14.1 min, cyanidin 16.3 min, petunidin 17.1 min, pelargonidin 18.3 min, and malvidin 19.2 min.

The elution order of anthocyanins depends on the polarity of the molecules, which is primarily affected by the anthocyanidin constituent, the number and type of attached sugar groups, as well as by any attached acyl groups [39,40]. Individual anthocyanins derived from the same anthocyanidins can have different elution orders if different sugar groups are attached [39]. Therefore, anthocyanin content and composition in the present study was determined following acid hydrolysis in order to convert the anthocyanins into anthocyanidins before identification and quantification by high-performance liquid chromatography with photodiode array detector (HPLC–PAD). Representative chromatograms are shown in Figure 7A,B for blueberry pomace water extracts and 50% ethanolic extracts, respectively. The USAE extraction conditions used to prepare these extracts were 40 °C–60 min, pH 5.0 and solid/liquid ratio 1/20 in water, and 40 °C–40 min, pH 6.3 and solid/liquid ratio 1/15 in 50% ethanol, respectively. Chromatograms corresponding to commercial standards (delphinidin-cyanidin-pelargonidin-malvidin mixed and petunidin) are displayed in Figure 7C,D, respectively.

As can be seen in Figure 7A,B, four individual anthocyanidins were identified in the blueberry pomace extracts. Water extracts and ethanolic extracts showed similar anthocyanidin profiles qualitatively speaking. The four anthocyanidins were present in all the extracts obtained in this study, regardless of the USAE conditions investigated. They are delphinidin (elution time 14.15, 14.145 and 14.166 min), cyanidin (16.316, 16.314 and 16.335 min), petunidin (17.111, 17.109 and 17.264 min) and malvidin (19.187, 19.188 and 19.199 min), with elution times in water extracts, ethanolic extracts and standards, respectively. Anthocyanidins follow this elution series (from shortest to longest elution times) because of differences in the hydroxyl and methoxy substituents (R1 and R2 groups, Figure 6) attached to the anthocyanidins. Delphinidin has the highest polarity because it contains the most hydroxyl groups, thus eluting first, while malvidin has the most methoxy groups, giving it a more hydrophobic character and making it the last to elute from a reverse phase column [40].

Malvidin was the most abundant anthocyanidin in both the water and ethanolic extracts, followed by dephinidin, petunidin and cyanidin. These four compounds have been previously detected in blueberries [5,38,39,40,41], but some authors also found peonidin in addition to the four other anthocyanins [38,39,40,41]. In the present study, peonidin was not detected and the four anthocyanins identified are in agreement with those extracted and identified by He et al. [5] from blueberry wine pomace subjected to USAE. With the exception of peonidin, the relative abundances of individual anthocyanins in our work is consistent with the proportions reported by Li et al. [38] in different blueberries cultivars produced in China, specifically, malvidin (41.0%), delphinidin (33.1%), petunidin (17.3%), cyanidin (7.1%) and peonidin (1.35%) [38]. In contrast, Wang et al. [41] found that cyanidin was the most abundant anthocyanin in different blueberry varieties, followed by peonidin, malvidin, delphinidin and petunidin. These variations in blueberry anthocyanin composition may be due to different blueberry varieties, agricultural practices, growing conditions, extraction conditions as well as analytical methods. Barnes et al. advised particular caution when identifying individual anthocyanins due to their high degree of structural complexity and similarity. Possible degradation of some anthocyanins when strong acids are used may also hamper their identification [40]. 

## 3. Materials and Methods

### 3.1. Plant Material and Chemicals

Blueberry pomace powder prepared by freeze-drying blueberry wine pomace (from *Vaccinium angustifolium*, lowbush blueberry) was kindly provided by Nova Agri Inc. (Centreville, NS, Canada) and stored at −30 °C prior to use. All the chemicals were of analytical reagent grade. Sodium benzoate and hydrochloric acid were obtained from Sigma Scientific (Oakville, ON, Canada). Ethanol, methanol and formic acid of HPLC grade were purchased from Caledon Laboratories (Georgetown, ON, Canada). Folin–Ciocalteu’s phenol reagent (2 N), sodium carbonate, sodium nitrite, aluminium chloride, gallic acid (GA), 6-hydroxy-2,5,7,8-tetramethylchroman-2-carboxylic acid (Trolox), and 2,2-diphenyl-1-picrylhydrazyl (DPPH) were purchased from Sigma-Aldrich Chemical Company (St Louis, MO, USA). (+)-Catechin and Folin–Ciocalteu reagent were purchased from Fluka (Milwaukee, WI, USA). Anthocyanin standards in the form of anthocyanidins (cyanidin chloride, dephinidin chloride, malvidin chloride, pelargonidin chloride, peonidin chloride and petunidin chloride) and dimethyl sulfoxide were obtained from Indofine Chemical Company Inc. (Somerville, NJ, USA).

### 3.2. Ultrasound-Assisted Extraction (USAE)

Ultrasound-assisted extraction was performed in an ultrasonic cleaner bath (15.5 × 14 × 9 mm, Symphony 97043-932, VWR, Mississauga, ON, Canada) with a maximum operating power of 35 kHz and 64 W. Prior to USAE, a beaker half-filled with distilled water was heated to the desired extraction temperature with stirring, using an agitator hotplate equipped with a temperature probe (IKA RCT basic, Staufen, Germany, 0–1500 rpm, 0–350 °C) and the temperature was kept constant. Meanwhile, 2 g of blueberry pomace powder was poured into a 125 mL brown-coloured flask. Then, milliQ water or various ethanol-milliQ water ratios was added as the extraction solvent to reach the appropriate solid/liquid ratio and shaken for a few minutes. The flask was tightly closed to avoid solvent evaporation, then immersed by suspension into the beaker of distilled water for a few minutes so that the mixture reached the desired extraction temperature. The heated water was subsequently poured into the ultrasound bath and the flask with a weight ring was placed into the bath. USAE was carried out at maximum operating power (35 kHz) for a specified duration at the set temperature (Table 1). The treatment was conducted in batch mode without agitation and cooling system since preliminary experiments using distilled water without any materiel immersed showed no increase in temperature (data not shown). After extraction, the resulting extracts were centrifuged at 6000 rpm for 15 min at room temperature and filtered by vacuum filtration through a 45 µm Millipore polyvinylidene difluoride (PVDF) membrane. The filtrate was transferred into a 100 mL amber glass volumetric flask, wrapped with aluminium foil to prevent degradation of bioactive compounds, and concentrated by rotary evaporation under vacuum (Büchi Rotavapor RII, Rose Scientific Ltd., Essen, Germany) at 40 °C and 100 mbars for 20 min. The filtered extract was stored in a brown-coloured bottle at 4 °C until further analyses. The USAE parameters that were varied are sonication time, solid/liquid ratio and solvent composition (% ethanol in water), pH and temperature, according to the experimental scheme summarized in Table 1. All extractions were carried out in triplicate.

### 3.3. Chemicals Analyses of Extracts

All chemical analyses, except TAC which was determined by HPLC, were performed in a 96-well microplate reader Synergy 2 equipped with Gen5TM data analysis software (Biotek Instruments Inc., Winooski, VT, USA).

#### 3.3.1. Determination of Total Phenolic Content (TPC)

Total phenolic content of the blueberry pomace extracts was determined using the method of Folin–Ciocalteu following the procedure described by Tournour et al. [42] with slight modification. Briefly, 25 μL of either sample or standard properly diluted with milliQ water were transferred into appropriate wells. With a multichannel pipet, 125 μL of 0.2 N Folin–Ciocalteu’s reagent were added to each well, then the plate was swirled and incubated in the dark at room temperature. After 8 to 10 min, 125 μL of 7.5% sodium carbonate was added. The obtained solution was mixed thoroughly and incubated at room temperature for 30 min at least and no more than 60 min. Subsequently, the absorbance was recorded at 765 nm with a spectrophotometric microplate reader (Synergy HT Multi-Detection Microplate Reader, BioTek Instruments, Winooski, VT, USA). Absorbance was compared to a gallic acid standard curve (R^2^ = 0.999) to quantify TPC in the sample. The results were expressed as milligrams of gallic acid equivalents per gram of dry matter (mg GAE/g DM). Each standard and sample solution was analysed in triplicate.

#### 3.3.2. Determination of Total Flavonoid Content (TFC)

Total flavonoid content (TFC) of the extracts was determined according to the 96-well microplate method [43] with some modification. A volume of 110 μL of 0.066 M sodium nitrite (NaNO_2_) was added to each of the 96 wells and 25 μL of standard or properly diluted sample solution was added. The plate was gently swirled and incubated at room temperature for 5 min. Then, 15 μL of 0.75M aluminium chloride (AlCl_3_) solution was added to the mixture simultaneously in each of the wells using a multichannel pipet. The plate was swirled again and incubated at room temperature. After 6 min, 100 μL of 0.5 M NaOH were added. The precipitations formed were gently dissolved using the multichannel pipet by avoiding the generation of air bubbles. Finally, absorbance was measured at 510 nm in the plate reader. All samples and standards were prepared in methanol and measured against a methanol reagent blank using the template of the microplate. Catechin (15–500 μg/mL) was used as a standard to generate a linear calibration curve (R^2^ = 0.998) and results were expressed as milligrams of catechin equivalents per gram of DM (mg CE/g DM). Each standard and sample solution was analysed in triplicate.

#### 3.3.3. Determination of Total Anthocyanin Content (TAC) and Identification of Anthocyanins

Total anthocyanin content (TAC) and individual anthocyanins were determined by HPLC-PAD using an Agilent 1100 series system equipped with a photodiode-array detector 200–800 nm (Agilent Technologies, Waldbronn, Germany). The column was a C-18 HPLC column, 5 μm, 150/4.6 mm (YMC Inc., Wilmington, NC, USA). The elution solvents were (A) 10% formic acid/milliQ water (*v*/*v*) and (B) 100% methanol. Solvent gradient was linear from 95% A/5% B to 40% A/60% B (0–20 min), isocratic at 40% A/60% B (20–23 min), linear from 40% A/60% B to 95%A/5% B (23–24 min), and isocratic at 95%A/5% B (24–28 min, run time 28 min). The detection wavelength was 520 nm. Flow rate was 0.7 mL/min, column temperature 25 °C, pressure 300 bars, sample temperature was ambient and injection volume was 40 μL.

Commercially available anthocyanidin standards of cyanidin chloride, delphinidin chloride, malvidin chloride, pelargonidin chloride, peonidin chloride and petunidin chloride were separately dissolved in 2 mL of dimethyl sulfoxide (99.9%) and used as standard stock solutions. The stock solutions were diluted in methanol (*v*/*v*) to prepare 3.125, 6.25, 12.5, 25.0 and 50 μg/mL solutions for all standards. For identification of the anthocyanins present in the extracts, these six standard solutions were separately injected into the column. 

TAC of the extracts were quantified after acid hydrolysis, which enables the determination of the aglycon forms of the anthocyanins (i.e., the anthocyanidins) (Figure 6). A 60 μL sample was transferred into a 50 mL flat-bottom centrifuge tube and 3 mL of milliQ water were added. The tube was capped and the sample was vortexed for 60 s. Then, 3.3 mL of hydrochloric acid (HCl 5N) were added. The mixture was heated in a water bath (100 °C for 60 min), then cooled to room temperature under running tap water. It was subsequently filtered through a 0.25 μm PTFE membrane filter into an HPLC vial and analysed by HPLC-PAD. Two replicates per sample were prepared. Malvidin was used as a standard to generate a linear calibration curve (R^2^ = 0.997) and the results were expressed as milligrams of malvidin equivalents per gram of DM (mg ME/g DM). Standard and sample solutions were analysed in triplicate.

#### 3.3.4. Determination of Antioxidant Activity

Antioxidant activity of the extracts was evaluated as DPPH free radical scavenging activity determined using the DPPH assay, as described by Herald et al. [43] with some modification. The DPPH stock solution (350 mM) was prepared daily in methanol and used to prepare the working solution (350 μM). Volumes of 225 μL methanol, 25 μL of methanol plus 200 μL of DPPH working solution, and 25 μL of standards or sample plus 200 μL of DPPH were respectively added to blank wells, control wells, and standard or sample wells using a multichannel pipet. The plate was sealed with sealing tape, gently swirled then incubated for 6 h at room temperature in the dark. After incubation, absorbance was recorded at 517 nm using the above-mentioned microplate reader. The percentage of DPPH quenched was calculated using Equation. 1:

% DPPH quenched = [1 − (A_sample_ − A_blank_)/(A_control_ − A_blank_)] × 100 (1)

where A is the absorbance of the sample, blank or control. Trolox (62.5–1000 μM) was used as a standard to generate a calibration curve (R^2^ = 0.998) and DPPH free radical scavenging activity was expressed as trolox equivalents (mg TE/g DM).

### 3.4. Statistical Analyses

Descriptive statistics were calculated and expressed as means ± standard deviation (SD). After checking for normality, means were compared using either one-way analysis of variance (ANOVA) followed by Tukey’s multiple comparison test, or the Kruskal–Wallis test followed by the Dunn’s multiple comparison test, as appropriate. Analyses were performed using Statistica version 7. Statistical significance was established at *p* ≤ 0.05.

## 4. Conclusions

The present study showed that the efficiency of ultrasound-assisted extraction of phenolic compounds from blueberry pomace is significantly influenced by the proportion of ethanol in the aqueous solvent, solid/solvent ratio, extraction temperature, sonication time and pH. The antioxidant activity of the extracts was also significantly affected. A binary solvent system (50% ethanol/water) was more efficient for extracting total phenolics, flavonoids and anthocyanins from blueberry pomace. In addition, decreasing the solid/solvent ratio led to superior polyphenol contents and antioxidant activity of the extracts. With 50% ethanol, higher temperature increased the total flavonoid content and antioxidant activity, but lowered the total phenolic content. USAE under slightly basic pH conditions positively affected total phenolic content and antioxidant activity compared to acidic pH, but lowered the anthocyanin content. Longer sonication time in water increased the anthocyanin content. The anthocyanin profiles of the ethanolic and aqueous extracts were qualitatively similar and consisted of malvidin, delphinidin, petunidin and cyanidin by decreasing order of relative concentration. These findings indicate that USAE is a method of choice for extracting high-value bioactive phenolics from blueberry pomace. For superior antioxidant activity of the extracts, the following USAE conditions are recommended: binary solvent system (50% ethanol/water), low solid/ethanolic solvent ratio, slightly basic pH and temperature above 20 °C. Selective enrichment of different phenolic fractions is possible under select USAE conditions. These findings are helpful for the valorisation of blueberry pomace using USAE as a green technology to produce health-promoting phenolic compounds.

## Figures and Tables

**Table 1:** Extraction conditions for ultrasound-assisted extraction of phenolic compounds from blueberry pomace.

	Solid/Liquid Ratio	Temperature (°C)	Time (min)	pH	Ethanol (% *v*/*v*) in Water
Runs 1–3 (Figure 1)	1/20	40	30–60–90	5.0	0
Runs 4–8 (Figure 2A)	1/10–(1/10) × 2 *–1/20–1/40	40	60	3.3	0
Runs 9–11 (Figure 2B)	1/10–1/15–1/20	40	60	3.3	50
Runs 12–14 (Figure 3)	1/15	40	40	3.3	10–50–90
Runs 15–17 (Figure 4)	1/15	40	40	3.3–6.3–8.3	50
Runs 18–20 (Figure 5)	1/15	20–40–60	40	3.3	50

* (1/10) × 2: two sequential extractions (1/10 for 30 min each time). All the experiments were conducted in triplicate.
